# Hypochromic red cells as predictors of anemia in patients undergoing hemodialysis: an observational retrospective study

**DOI:** 10.1038/s41598-021-03746-2

**Published:** 2021-12-20

**Authors:** Youn Kyung Kee, Hee Jung Jeon, Jieun Oh, Dong Ho Shin

**Affiliations:** Department of Internal Medicine, College of Medicine, Hallym University, Kangdong Sacred Heart Hospital, 150, Seongan-ro, Gangdong-gu, Seoul, 05355 Korea

**Keywords:** Medical research, Nephrology

## Abstract

The percentage of hypochromic red blood cells (%HRC) estimates the availability of iron by evaluating the degree of hemoglobinization. We investigated whether %HRC was a predictor of anemia in patients undergoing hemodialysis. We recruited 142 patients undergoing routine hemodialysis between 2017 and 2019. Delta hemoglobin level (ΔHb)_1mo-baseline_ was calculated as the difference between the hemoglobin level at 1 month after study enrollment and that at the time of study enrollment. Development of anemia was defined as hemoglobin level ≤ 15% of baseline. The median %HRC was 3.1%. There was a significant negative correlation between (ΔHb)_1mo- baseline_ and %HRC (r =  − 0.63, *P* < 0.001). The incidence of anemia was significantly higher in patients with %HRC > 3.1% than in those with %HRC ≤ 3.1%. In the multivariate logistic regression analysis, %HRC was significantly related to the development of anemia (odds ratio 2.57, 95% confidence interval [CI] 1.72–3.85, *P* < 0.001). The best cutoff value for %HRC to predict the development of anemia was 4.3%, with a sensitivity and specificity of 67.74 (95% CI, 54.7–79.1) and 97.50 (95% CI, 91.3– 99.7), respectively. %HRC is an independent predictor of anemia in patients undergoing hemodialysis. %HRC ≤ 4.3% is an early marker to consider changing the anemia treatment.

## Introduction

Anemia is a common complication in patients undergoing hemodialysis due to a decreased production of erythropoietin or iron deficiency^1,2^. This condition exacerbates fatigue, exercise intolerance, depression, and dyspnea and increases morbidity and mortality related to cardiovascular disease in these patients^3–5^. It is usually treated with erythropoiesis-stimulating agents (ESAs) and/or extra iron. However, in many studies on the optimal hemoglobin levels in these patients, higher hemoglobin levels were associated with the development of hypertension and an increased clotting tendency, eventually leading to adverse cardiovascular outcomes^6–8^. Clinical experts suggest that optimal hemoglobin levels are usually in the range of 10–11.5 g/dL^9^. However, optimal hemoglobin levels for patients receiving treatment with ESAs and/or extra iron are not well defined. To achieve the optimal hemoglobin levels and the maximum benefit of ESAs, adequate iron storage is essential, as decreased iron storage is the most common cause of resistance to ESAs.

Bone marrow biopsy is the most accurate way to measure iron storage^10^. However, it is invasive and difficult to apply to patients undergoing hemodialysis. Thus, the status of iron storage is commonly estimated through the biochemical markers of iron metabolism, such as serum iron, serum ferritin, and transferrin saturation (TSAT)^11,12^. However, there have been concerns that these markers do not exactly reflect the status of iron storage^11^. Particularly, ferritin, an acute phase reactant, is often elevated in the serum of patients undergoing hemodialysis, regardless of their iron stores^11^. Additionally, ESA-induced functional iron deficiency is common in these patients^13^, which occurs when sufficient circulating iron is incorporated into erythroid precursors and cannot be released from iron storage^12,13^. Consequently, these patients commonly have low TSAT levels and normal or high serum ferritin levels^10^.

Recently, several markers that directly estimate the availability of iron by evaluating the degree of hemoglobinization have been developed. Among them, the reticulocyte hemoglobin content (CHr) and the percent hypochromic red blood cells (%HRC) are the most promising markers^14–16^. These markers estimate the hemoglobin content of red blood cells and not the amount of iron storage. These markers are more accurate than serum iron, serum ferritin, or TSAT in diagnosing iron deficiency among patients undergoing hemodialysis^15–17^. However, data on the clinical usefulness of %HRC in predicting the development of anemia is lacking. Therefore, in this study, we aimed to investigate whether %HRC is a predictor of anemia in patients undergoing hemodialysis.

## Results

### Study population

**Figure 1:**
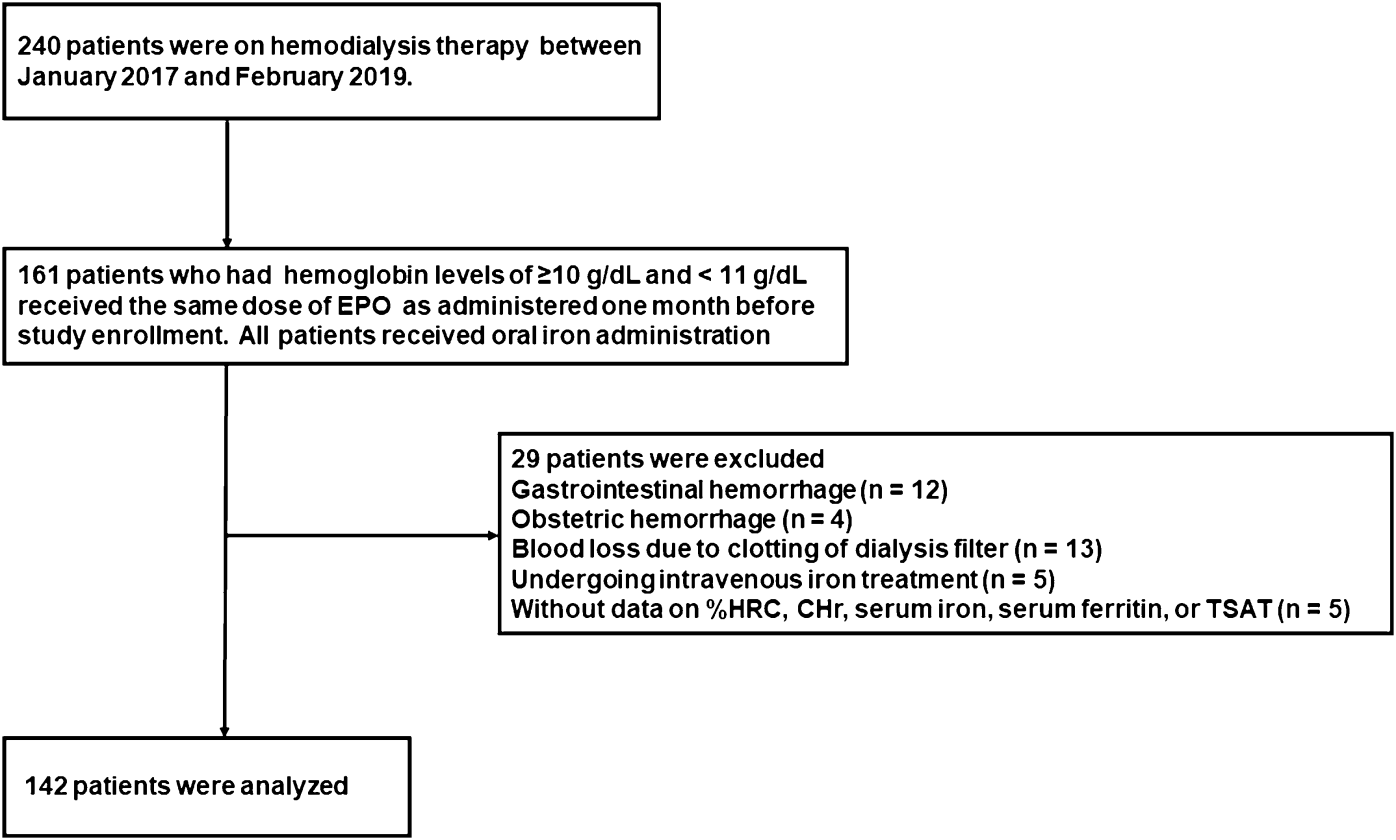
Flowchart of study population enrollment.

A total of 240 patients received hemodialysis therapy between October 2017 and September 2019. Among them, 161 patients were eligible for the study. Twenty-nine patients were excluded due to bleeding events such as gastrointestinal hemorrhage (n = 7), obstetric hemorrhage (n = 4), and blood loss due to clotting of the dialysis filter (n = 8) during the study observation period. Additionally, patients undergoing intravenous iron treatment (n = 5) and those without data on %HRC, CHr, serum iron, serum ferritin, or TSAT (n = 5) were excluded. Thus, a total of 142 patients undergoing hemodialysis were included in the analysis (Fig. 1).

### Baseline characteristics of patients

**Figure 2:**
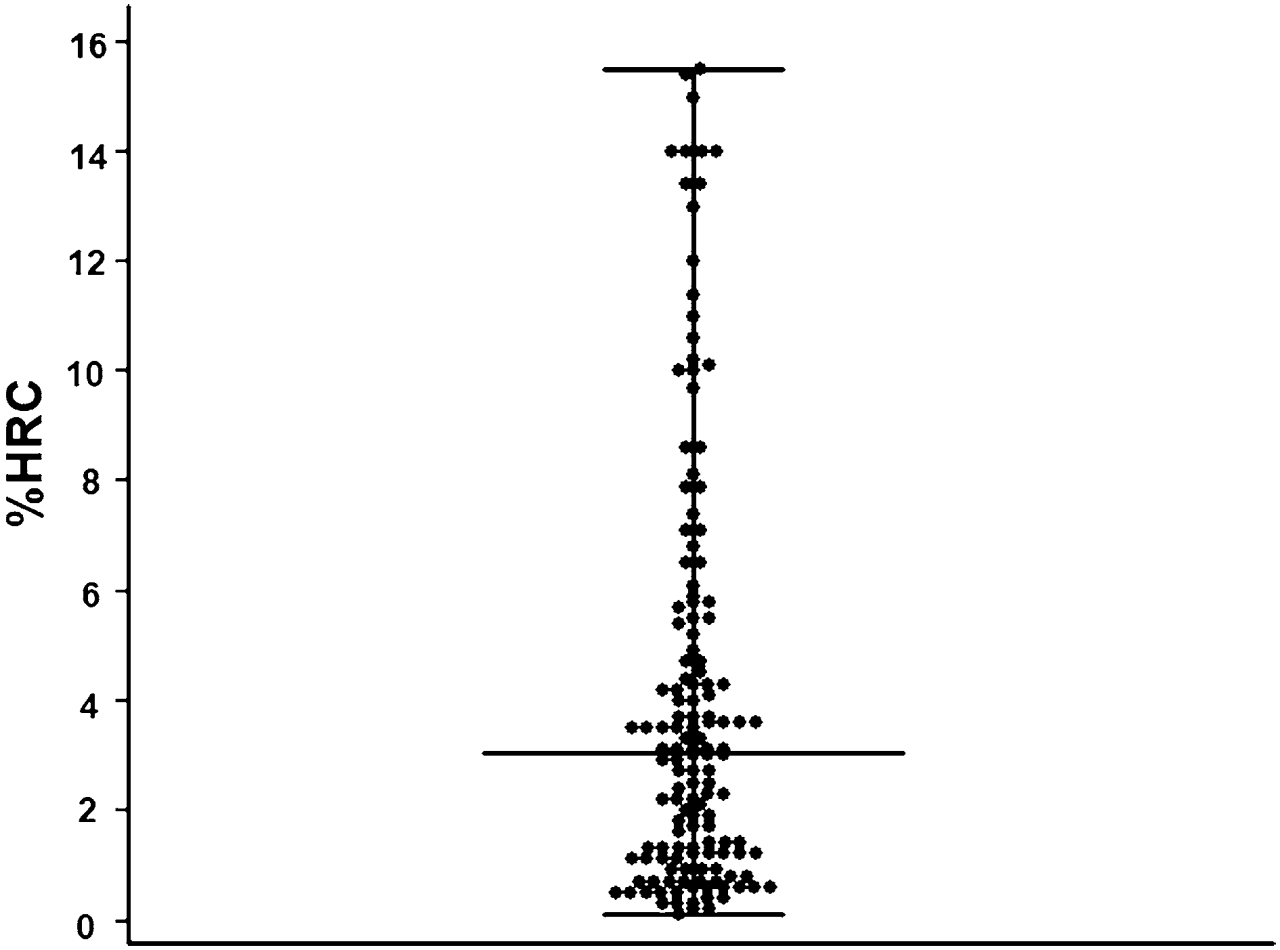
Scattered plots of percent hypochromic red cells. The bars and error bars indicate the medians and ranges, respectively.

The demographic, clinical, and biochemical data of the patients are shown in Table 1. Among the 142 patients, 80 (56.3%) were men; the median age was 65 years, median dialysis duration before study enrollment was 51.8 months, and underlying cause of end-stage renal disease (ESRD) in 90 patients was diabetes (63.4%). The median %HRC was 3.1% (range, 0.5%–15.5%; Fig. 2). When patients were divided into two groups according to their median %HRC, the dialysis duration before study enrollment, iron concentration, and CHr were significantly higher in the patients with %HRC ≤ 3.1% than in those with %HRC > 3.1%. However, there were no differences between the groups with respect to age, male sex, underlying ESRD cause, medication use, and values of ferritin, TIBC, PTH, calcium, phosphate, C-reactive protein (CRP), TSAT, and Kt/V.

**Table 1:** Baseline characteristics of patients.

	Total (n = 142)	%HRC < 3.1% (n = 71)	%HRC ≥ 3.1% (n = 71)	*p-*value
**Demographic data**				
Age (years)	65 (58.0–73.0)	64.0 (56.0–71.5)	67.0 (60.5–74.0)	0.05
Male, n (%)	80 (56.3)	46 (64.8)	34 (47.9)	0.06
**Clinical data**				
Hemodialysis duration (months)	51.8 (7.5–77.4)	60.0 (14.7–90.1)	35.6 (5.4–64.1)	0.02
Underlying end-stage renal disease cause				
Diabetes, n (%)	90 (63.4)	48 (67.6)	42 (59.2)	0.38
Hypertension, n (%)	29 (20.4)	11 (15.5)	18 (25.4)	0.21
Glomerulonephritis, n (%)	9 (6.3)	5 (7.0)	4 (5.6)	0.73
Polycystic kidney disease, n (%)	4 (2.8)	1 (1.4)	3 (4.2)	0.61
Contrast-induced nephropathy, n (%)	8 (5.6)	6 (8.5)	2 (2.8)	0.28
Unknown, n (%)	2 (1.4)	0 (0.0)	2 (2.8)	0.48
Medication use				
Vitamin D analogs, n (%)	69 (48.6)	34 (47.9)	35 (49.3)	0.99
Calcium-based phosphate binder, n (%)	62 (43.7)	29 (40.8)	33 (4.5)	0.61
Non-calcium-based phosphate binder, n (%)	81 (57)	46 (64.8)	35 (49.3)	0.09
RASB	76 (53.5)	42 (59.2)	34 (47.9)	0.24
Darbepoetin alfa, n (%)^a^	126 (88.7)	65 (91.5)	61 (85.9)	0.43
Dose of darbepoetin alfa (μg/week)^b^	26 ± 12.7	26.3 ± 11.1	25.6 ± 14.2	0.743
**Laboratory parameters**				
Hemoglobin (g/dL)	10.4 (9.2–10.5)	10.3 (10.1–10.6)	10.4 (10.1 -10.6)	0.56
1mo follow-up hemoglobin (g/dL)	10.1 (9.6–10.4)	10.2 (10.0–10.6)	9.8 (8.7–10.2)	< 0.001
CHr (pg)	32.2 ± 2.1	32.6 ± 1.9	31.8 ± 2.3	0.03
Ferritin (ng/mL)	162.2 (89.3–277.8)	161.6 (96.9–251.7)	162.9 (87.2–282.5)	0.85
Iron (ug/dL)	62.5 (48.0–85.0)	69.0 (56.5–90.5)	57.0 (43.5–71.5)	0.01
TIBC (ug/dL)	231.5 (205.0–261.0)	244.0 (211.5–263.5)	223.0 (202.0 -254.5)	0.09
TSAT (%)	26.8 (20.7–35.1)	30.0 (21.5–36.5)	25.9 (19.3–32.5)	0.09
PTH (pg/mL)	244.7 (135.1–400.9)	253.5 (137.8–361.4)	244.7 (132.4–443.4)	0.56
Calcium (mg/dL)	8.8 (8.3–9.1)	8.8 (8.4–9.2)	8.7 (8.2–9.0)	0.08
Phosphate (mg/dL)	4.6 (3.7–5.5)	4.9 (3.8–5.7)	4.3 (3.5–5.2)	0.07
CRP (mg/L)	3.0 (2.0–4.0)	3.0 (2.0–4.0)	3.0 (2.0–5.0)	0.09
Kt/V	1.5 (1.3–1.6)	1.4 (1.3–1.5)	1.5 (1.3–1.6)	0.37

Values are expressed as means ± standard deviations, medians (interquartile ranges), or numbers (percentages).

%HRC, percent hypochromic red blood cells; *RASB* renin–angiotensin–aldosterone system blockade, *CHr* reticulocyte hemoglobin content, *TIBC* toral iron-binding capacity, *TSAT* transferrin saturation, *PTH* parathyroid hormone, *CRP* C-reactive protein.

^a^Information at the time of study enrollment.

^b^Information between the time of study enrollment and 1 month after study enrollment.

### Correlation between ΔHb and other variables

**Figure 3:**
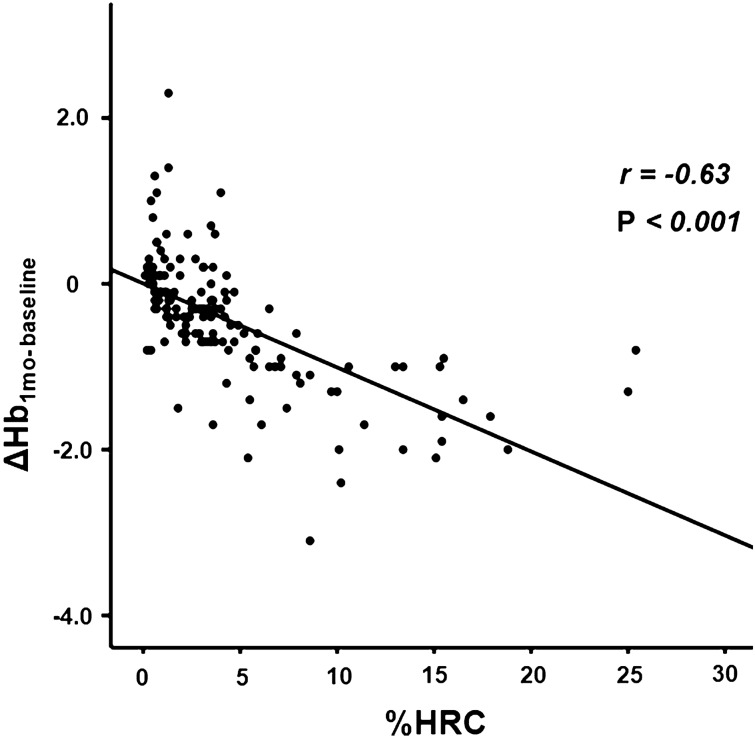
Relationship between ΔHb_1mo- baseline_ and %HRC. Data are presented as correlation coefficients (*r*) and *p*-values. ΔHb_1mo- baseline_ is calculated as hemoglobin value at 1 month after study enrollment—hemoglobin level at study enrollment. %HRC, percent hypochromic red cells.

There was a significant negative correlation between the delta hemoglobin level (ΔHb_1mo- baseline)_ and %HRC (r =  − 0.63, *P* < 0.001; Fig. 3). Moreover, despite a significant positive correlation between ΔHb_1mo- baseline_ and CHr (r = 0.21, *P* = 0.01), there were no correlations between ΔHb_1mo- baseline_ and the concentrations of iron, ferritin, and TSAT.

### Development of anemia according to %HRC

Among the 142 patients, 62 (43.7%) developed anemia at 1 month after study enrollment. The incidence of anemia was significantly higher in patients with %HRC > 3.1% than in those with %HRC ≤ 3.1% (70.4% vs. 6.9%, P < 0.001). However, hemoglobin levels were significantly lower in patients with %HRC > 3.1% than in those with %HRC ≤ 3.1% [9.8 (9.0 0–10.1) vs. 10.2 (10.0–10.6), *P* < 0.001] at 1 month after study enrollment.

### Variables related to the development of anemia

The variables related to the development of anemia are shown in Table 2. In univariate analysis, %HRC and CHr were found to be significant predictors of the development of anemia. Moreover, age, male sex, use of non-calcium-based phosphate binders, and CRP levels were significantly related with the development of anemia. In contrast, the concentrations of ferritin, iron, and TSAT were not associated with the development of anemia. Specifically, %HRC remained to be a significant predictor of anemia, even after adjusting for potential confounding factors related to the development of anemia.

**Table 2:** Variables related to the development of anemia.

Variable	Univariate analysis		Multivariate analysis	
	OR (95% CI)	*p*-value	OR (95% CI)	*p*-value
Age (per year)	1.03 (0.99–1.07)	0.14	1.05 (0.98–1.12)	0.15
Male (vs. Female)	0.24 (0.11–0.56)	0.001	0.50 (0.14–1.89)	0.51
HD duration (per 1 month)	0.99 (0.98–1.00)	0.03	1.00 (0.99–1.01)	0.5
Non-calcium-based phosphate binder	0.39 (0.18–0.87)	0.02	0.80 (0.16–3.97)	0.78
RASB	0.90 (0.41–1.96)	0.79	2.41 (0.74–7.80)	0.14
Dose of darbepoetin alfa (per 10 μg/week)	0.91 (0.67–1.24)	0.56	0.88 (0.53–1.46)	0.62
Hemoglobin (per 1 g/dL)	1.33 (035–5.04)	0.67	8.58 (0.96–76.9)	0.06
%HRC (per 1%)	2.11 (1.61–2.76)	< 0.001	2.57 (1.72–3.85)	< 0.001
CHr (per 1 pg)	0.79 (0.65–0.95)	0.01	1.06 (0.77–1.45)	0.72
Ferritin (per 1 ng/mL)	1.00 (0.99–1.00)	0.44	1.00 (0.99–1.01)	0.95
Iron (per 1 ug/dL)	0.99 (0.98–1.00)	0.12	1.06 (0.98- 1.15)	0.12
TIBC (per 1 ug/dL)	0.99 (0.99–1.01)	0.9	0.98 (0.95–1.00)	0.08
TSAT (per 1%)	0.99 (0.96–1.01)	0.31	0.91 (0.77–1.07)	0.25
PTH (per 1 pg/mL)	1.00 (1.00–1.01)	0.15	1.00 (0.99–1.00)	0.99
Phosphate (per 1 mg/dl)	0.87 (0.66–1.13)	0.29	1.10 (0.54–2.20)	0.8
CRP (mg/L)	1.02 (1.00–1.03)	0.03	1.11 (0.91–1.35)	0.3
Kt/V (per 0.1)	1.19 (0.99–1.43)	0.06	0.86 (0.64–1.16)	0.32

Values are expressed as mean ± standard deviation, median (interquartile range), or number (percentage).

*%HRC* percent hypochromic red blood cells, *RASB* renin–angiotensin–aldosterone system blockade, *CHr* reticulocyte hemoglobin content, *TIBC* toral iron-binding capacity, *TSAT* transferrin saturation, *PTH* parathyroid hormone, *CRP* C-reactive protein, *OR* odds ratio, *CI* confidence interval.

### Comparison of variables to predict the development of anemia

**Figure 4:**
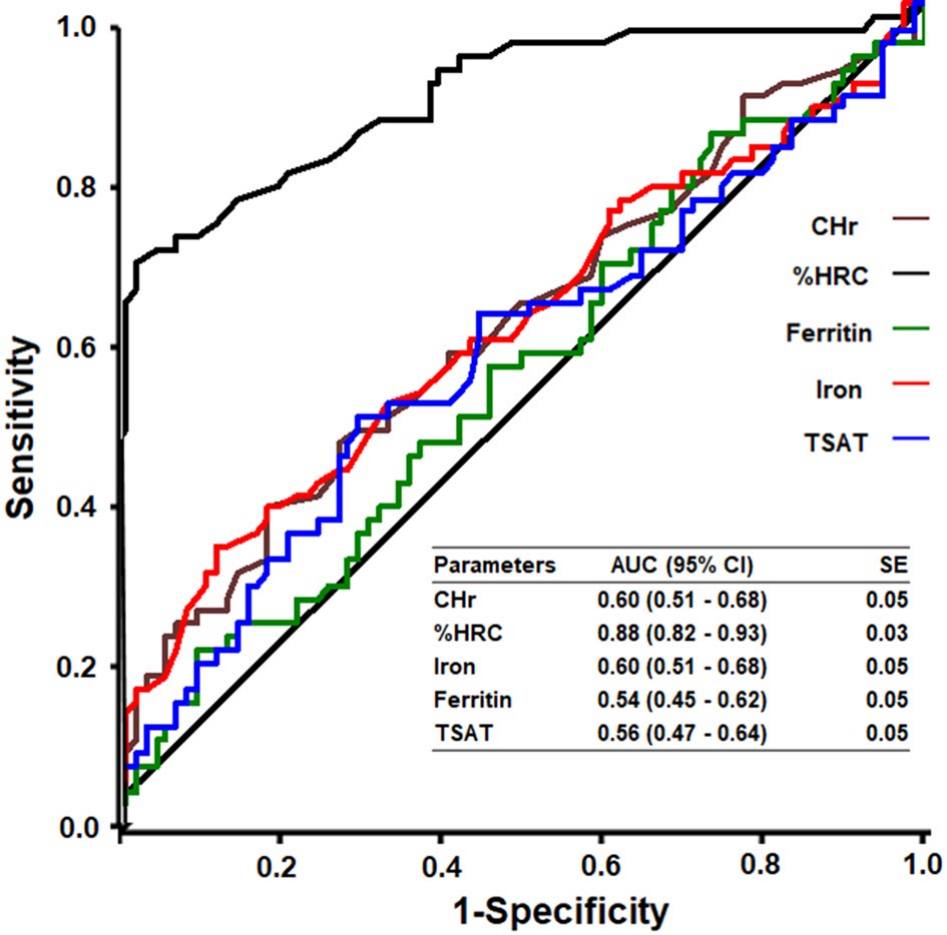
Receiver operating characteristic (ROC) curves for CHr, %HRC, iron, ferritin, and TSAT with respect to the prediction of anemia. CHr, reticulocyte hemoglobin content; %HRC, percent hypochromic red cells; TSAT, transferrin saturation.

To compare the ability of iron, ferritin, TSAT, %HRC, and CHr to predict the development of anemia, receiver operating characteristic (ROC) curve analysis with the area under the ROC curve (AUC) was performed. The AUC of %HRC was significantly larger than that of the other variables. ROC curve analysis with AUC was also used to obtain the optimal cutoff value of %HRC to predict the development of anemia. The best cutoff value for %HRC to predict the development of anemia was 4.3%, with a sensitivity and specificity of 67.74 (95% confidence interval [CI], 54.7–79.1) and 97.50 (95% CI, 91.3–99.7), respectively (Fig. 4).

## Discussion

This retrospective observational study of patients undergoing hemodialysis showed that an increase in %HRC was associated with a decrease in hemoglobin levels in the future. Particularly, %HRC was found to be an independent predictor of anemia, defined as hemoglobin level ≤ 15% of baseline.

Inflammation, inadequate dialysis, hyperparathyroidism, and use of angiotensin receptor blockers or angiotensin-converting enzyme inhibitors are associated with ESA-resistant anemia in patients undergoing hemodialysis^18^. Additionally, ESA-induced functional iron deficiency is often observed in these patients^13^. When the iron metabolism is abnormal, an imbalance between the iron requirement of the erythroid precursors and the iron supply to the erythroid precursors occurs in the bone marrow^12^. Eventually, it prevents the erythrocytes from maintaining at a sufficient rate to allow normal hemoglobinization and reduce the hemoglobin content of reticulocytes and erythrocytes. Traditionally, the standard biochemical markers of iron metabolism include serum iron, serum ferritin, and TSAT^11,12^. However, evaluating iron metabolism using these markers in patients undergoing hemodialysis is difficult. To overcome this limitation, modern hematology analyzers using flow cytometry of individual erythrocytes or reticulocytes have been developed^19^.

Reticulocytes last only 1–2 days in the serum before developing into mature red blood cells (RBCs). Therefore, when these are first released from the bone marrow, the CHr can reflect the amount of iron immediately available for erythropoiesis. Reticulocytes in samples stored at room temperature remain stable overtime, indicating no time constraints to be analyzed within a few hours after blood sampling. Meanwhile, when RBCs are continuously produced in the bone marrow under iron-restricted erythropoiesis, hemoglobin synthesis becomes severely impaired, resulting in the production of erythrocytes with low hemoglobin content or hypochromic RBCs. Iron-restricted erythropoiesis can be detected by measuring the percentage of erythrocytes with hemoglobin concentration < 280 g/L. %HRC is related to the iron status over several months because erythrocytes have a life span of approximately 120 days. The erythrocytes in samples stored at room temperature tend to swell progressively; therefore, they must be analyzed within 6 h to measure %HRC^2,12,14,15,20^.

Several studies have suggested that %HRC and CHr are effective parameters for reflecting the iron status of patients undergoing hemodialysis^15–17,21^. Nicola et al. showed that the best available marker to identify iron responders was %HRC > 6%^21^. In their study, a patient receiving erythropoietin was defined an iron responder when there is an increase in hemoglobin levels ≥ 15% of baseline after intravenous iron administration. Additionally, an increase of 15% in the hemoglobin levels of iron responders usually occurred 1–3 months after study enrollment^21^. Based on these findings, the NICE guideline recommends measuring the %HRC or CHr to diagnose iron deficiency or determine the potential responsiveness to iron in patients undergoing hemodialysis. In this guideline, the thresholds to diagnose iron deficiency are %HRC > 6% and CHr < 29 pg^22^. In the present study, we showed that %HRC was the only independent predictor of anemia at 1 month after study enrollment. Given that %HRC is a late indicator of iron-restricted erythropoiesis, this finding is reasonable. Interestingly, univariate analysis demonstrated the association between the development of anemia and %HRC but not hemoglobin. Hemoglobin tests to diagnose anemia cannot evaluate iron metabolism. Even if the baseline hemoglobin level in this study was low, if the amount of iron available for erythropoiesis was sufficient, anemia might not occur at 1 month after study enrollment. Meanwhile, multivariate logistic regression analysis of this study showed that CHr was a weak confounder of the association between %HRC and the development of anemia at 1 month after study enrollment. Considering that CHr is an early indicator of iron-restricted erythropoiesis, this result could be explainable. Finally, in this study, the best cutoff value for %HRC to predict the development of anemia was 4.3%, which is lower than the 6% suggested by the guideline to diagnose iron deficiency.

Our study has several limitations. First, this was a retrospective, observational, single-center study, using repeated measures data based on a small number of patients undergoing hemodialysis. Therefore, regression to the mean might occur in this study design. Second, the hemodialysis centers measured %HRC, CHr, and standard biochemical markers of iron metabolism in patients undergoing hemodialysis every 3 months. Therefore, the change in these markers could not be evaluated 1 month after study enrollment. Third, due to the study design, it was not possible to investigate the changes in %HRC after intravenous iron administration, addition, or increase in ESA.

Despite these limitations, the present study shows that %HRC is an independent predictor of anemia development in patients undergoing hemodialysis. Although %HRC ≤ 4.3% is an early marker to consider changing the anemia treatment in this study, further well-designed clinical studies are needed to clarify this. Especially, to control residual confounder factors, a self-comparison between baseline %HRC and the ΔHb1mo-baseline in one patient would be a good study design.

## Methods

### Ethics statement

The study protocol was approved by the Institutional Review Board of Kangdong Sacred Heart Hospital (Refs. Kangdong 2021-08-002). This study was performed in accordance with the Declaration of Helsinki. In this retrospective medical record-based study, de-identified participant data were used; therefore, the requirement for a written informed consent was waived. Of note, the full name of ethics committee that approved the waiver for informed consent is Kangdong Sacred Heart Hospital IRB.

### Patients

This observational retrospective study was performed at the Dialysis Clinic of Kangdong Sacred Heart Hospital. Eligible patients underwent hemodialysis thrice a week as outpatients between October 2017 and September 2019, had hemoglobin levels of 10–11 g/dL, and received the same dose of ESA 1 month before study enrollment. All patients underwent hemoglobin testing every month. Depending on the results, a nephrologist decided whether to administer and adjusted the doses of ESA. All patients received darbepoetin alfa in cases when an ESA administration was needed. Once the doses of darbepoetin alfa was determined, administration was performed every week until the next month.

In contrast, patients who had blood loss due to gastrointestinal hemorrhage, obstetric hemorrhage, or clotting of the dialysis filter during the study observation period and those who did not have data on %HRC, CHr, serum iron, serum ferritin, or TSAT were excluded. Additionally, patients receiving intravenous iron treatment from 1 month before study enrollment to 1 month after study enrollment were excluded to rule out the effect of intravenous iron treatment. Of note, all enrolled patients received oral ferrous sulfate as 256 mg tablets (80 mg of elemental iron) twice per day from 1 month before study enrollment to 1 month after study enrollment. The pills were consumed between meals, and enteric coated formulations were avoided.

### Hemodialysis

All patients received hemodialysis using an SDS-20 dialysis machine (JMS Co., Ltd., Japan). Each hemodialysis treatment lasted for 4 h using a dialyzer with a blood flow rate of 250–300 mL/min and a dialysate flow of 500 mL/min.

### Data collection

Data on baseline characteristics, including demographic, clinical, and laboratory parameters, were obtained from medical records. Laboratory parameters were measured using ADVIA 2120 Hematology System (Siemens Healthcare Diagnostics, Tarrytown, NY, Germany), Beckman Coulter AU5800 (Beckman Coulter Inc., Brea CA, USA), and ADVIA Centaur XP (Siemens Healthcare Diagnostics, Erlangen, Germany). The blood samples drawn from each patient were immediately transported to the chemical laboratory department at room temperature, and the assay was conducted within 1 h of blood sampling.

### Outcome assessment

The study endpoint was evaluated using ΔHb. ΔHb_1mo-baseline_ was calculated as the difference between the hemoglobin level at 1 month after study enrollment and that at study enrollment. Development of anemia was defined as hemoglobin level ≤ 15% of baseline.

### Statical analyses

Statistical analyses were performed using SPSS (version 19.0; SPSS Inc., Chicago, IL, USA). The Kolmogorov–Smirnov test was used to analyze the normality of the variable distribution. Continuous variables are expressed as means ± standard deviations or median and interquartile, and categorical variables are expressed as numbers (percentages). Differences between two groups were assessed using Student’s t-test, the χ^2^, or Fisher’s exact test. Of note, since the data of %HRC were not normally distributed, the median value of 3.1 in %HRC was selected to divided patients into two groups. Pearson’s correlation analysis was performed to elucidate the relationship between %HRC and ΔHb. The predictive value for the development of anemia was analyzed using ROC curve analysis, which calculates AUC. The analysis of independent predictive variables for the development of anemia was ascertained by multivariate logistic regression analysis, which included all covariates with *p*-values < 0.1 in univariate analysis. Although *p*-values were ≥ 0.1, potential confounding factors were included in the multivariate analysis.

## Data Availability

The datasets generated during and/or analyzed during the current study are available from the corresponding author on reasonable request.
